# Dopamine mediates vagal modulation of the immune system by electroacupuncture

**DOI:** 10.1186/cc14057

**Published:** 2014-12-03

**Authors:** R Torres Rosas, P Morcillo, L Ulloa

**Affiliations:** 1 Medical Research Unit on Immunochemistry, National Medical Center Siglo XXI, Mexico City, Mexico; 2 Laboratory of Anti-inflammatory Signaling, Department of Surgery, Rutgers University New Jersey Medical School, Newark, NJ, USA; 3 Center of Immunology and Inflammation, Rutgers University New Jersey Medical School, Newark, NJ, USA

## Introduction

Previous anti-inflammatory strategies against sepsis, a leading cause of death in hospitals, had limited efficacy in clinical trials, in part because they targeted single cytokines and the experimental models failed to mimic clinical settings [1-3]. Neuronal networks represent physiological mechanisms, selected by evolution to control inflammation, that can be exploited for the treatment of inflammatory and infectious disorders [3].

## Methods

Animal procedures were approved by the Institutional Animal Care & Use Committee of the New Jersey Medical School of Rutgers University. All animal experiments were performed in 6-week-old to 8-week-old (~25 ± 5 g) male mice without any exclusion criteria. Experimental sepsis: endotoxemia and CLP were performed as we previously described. LPS was dissolved in sterile pyrogen-free PBS and sonicated for 30 minutes immediately before use. Mice received a LD_50 _dose of LPS (6 mg/kg body weight i.p.). LPS was added to the whole blood to a final concentration of 250 ng/ml for the *in vitro *procedures. Selective neurectomies and electrical stimulations: all selective neurectomies and electrical stimulations were performed in mice anesthetized with ketamine and xylazine. The electrical stimulation in electroacupuncture and direct nerve stimulation (sciatic and vagus nerves) was performed with a continuous-mode stimulation for 15 minutes with a electrical potential difference of 4 V, an electric current of 40 mA, a pulse width of 50 μs and a frequency of 10 Hz using an electrostimulator.

## Results

Here, we report that sciatic nerve activation with electroacupuncture controls systemic inflammation and rescues mice from polymicrobial peritonitis. Electroacupuncture at the sciatic nerve controls systemic inflammation by inducing vagal activation of aromatic L-amino acid decarboxylase, leading to the production of dopamine in the adrenal medulla. Experimental models with adrenolectomized mice mimic clinical adrenal insufficiency [4], increase the susceptibility to sepsis and prevent the anti-inflammatory effects of electroacupuncture. Dopamine inhibits cytokine production via dopamine type 1 (D1) receptors. D1 receptor agonists suppress systemic inflammation and rescue mice with adrenal insufficiency from polymicrobial peritonitis. Our results suggest a new anti-inflammatory mechanism mediated by the sciatic and vagus nerves that modulates the production of catecholamines in the adrenal glands.

## Conclusion

From a pharmacological perspective, the effects of selective dopamine agonists mimic the anti-inflammatory effects of electroacupuncture and can provide therapeutic advantages to control inflammation in infectious and inflammatory disorders. Preliminary results in human clinical trials indicate that electroacupuncture attenuates the postsurgical inflammatory response decreasing the serum levels of inflammatory cytokines.
